# Digital Detection of Suicidal Ideation: A Scoping Review to Inform Prevention and Psychological Well-Being

**DOI:** 10.3390/bs15121601

**Published:** 2025-11-21

**Authors:** Benedetta Trentarossi, Mateus Eduardo Romão, Serena Barello, Makilim Nunes Baptista, Silvia Damiana Visonà, Giacomo Belli

**Affiliations:** 1 Department of Public Health, Experimental and Forensic Medicine, University of Pavia, 27100 Pavia, Italy; 2 WHYpsy Lab, Department of Brain and Behavioral Sciences, University of Pavia, 27100 Pavia, Italyserena.barello@unipv.it (S.B.); 3 Unit of Applied Psychology, IRCCS Mondino Foundation, 27100 Pavia, Italy; 4 Department of Psychology at Pontifical Catholic University of Campinas, State University of Campinas, Campinas 13034-685, Brazil

**Keywords:** suicide prevention, scoping review, suicidal ideation, online, digital suicide

## Abstract

Suicide is a major global public health concern, especially among young people. Given that digital surroundings are progressively influencing communication patterns, young people frequently communicate their feelings online, including suicidal thoughts. By promptly drawing attention to these posts, a crucial preventive measure could be taken. A scoping review guided by the research question “What is the current state of the art in detecting suicidal ideation in online posts?” following PRISMA guidelines. Out of the 1584 articles identified, only 48 met the inclusion criteria. The majority of articles were related to posts written in English on Reddit and Twitter. The main aim of the studies were interpretative (aim to explore how suicidal ideation is expressed in online environments) or predictive (aim to identify posts that may indicate suicidal ideation) and most of the posts were analyzed using artificial intelligence rather than traditional methods. Some, however, used mixed methods. Despite the potential of AI for rapidly processing and annotating suicidal notes, several hurdles remain, especially ethically obtained data sets and limited cross-cultural portability of models. Furthermore, current AI systems fail to interpret metaphors, irony, or context-specific meaning underscoring the requirement for hybrid models combining machine speed with human judgment.

## 1. Introduction

Suicide—the voluntary and intentional act of ending one’s own life—is a major global public health concern, accounting for about 700,000 deaths each year (X. Liu et al., 2019; World Health Organization, 2025). According to the World Health Organization (WHO), suicide ranks among the leading causes of death in young people aged 15 to 29, where it is the third most frequent cause (World Health Organization, 2025). This alarming phenomenon must be understood within a broader socio-cultural and technological context, in which communication practices are increasingly shaped by digital environments. Today, the immediacy of face-to-face dialogue is often replaced or complemented by the expression of thoughts and emotions through social media, forums, and other online platforms. Social media can offer support and connectivity, but it can also be associated with distress, pain, and a space for expressing these feelings and thoughts (Cai et al., 2023; Wu et al., 2016).

Adolescents and young adults, in particular, spend a significant portion of their time online, engaging with platforms that have become spaces not only for identity exploration and peer connection but also for the expression of emotional distress and suicidal ideation. From early platforms like MySpace or Facebook to more recent forums such as Reddit, Twitter, Tumblr, or Instagram, individuals experiencing suicidality have used the digital space to share personal struggles—including explicit expressions of suicidal thoughts and even suicide notes (Falcone et al., 2020; Huang et al., 2007). Unlike traditional handwritten notes discovered post-mortem, these digital messages can be visible in real time, opening a window for potential early intervention. However, it is important to note that not all online expressions occur in real time. In some cases, messages or suicide notes may be pre-scheduled or published posthumously.

Suicide notes—whether private or public—offer vital insight into the emotional states, interpersonal dynamics, and contextual triggers preceding suicidal acts (Karbeyaz et al., 2014). When these notes are shared online, they exist within a system of digital grief, collective witnessing, and social contagion (Walling, 2021). While such visibility may amplify risk through phenomena like the Werther effect—where reports of suicide may contribute to subsequent suicides (Wong & Li, 2017)—it also provides a unique opportunity to identify risk signals earlier and at scale (Niederkrotenthaler et al., 2020).

In recent years, digital platforms have started to implement tools aimed at identifying and reporting potentially high-risk content to emergency services, since the first research on digital phenotype (Birk & Samuel, 2022). These efforts have been bolstered by advances in artificial intelligence (AI), particularly in natural language processing (NLP), which offer automated systems capable of parsing large volumes of text and detecting linguistic patterns associated with suicidality (Belli et al., 2025).

Despite these advances, the current landscape of research is fragmented. Terminologies vary, platform-specific analyses abound, and few studies have attempted to integrate the multiple disciplinary perspectives—ranging from computer science and data ethics to clinical psychology and forensic medicine—necessary to build effective, ethical, and scalable detection systems. To fully understand how suicidal ideation is expressed online and how it can be responsibly identified, a comprehensive overview of the field is urgently needed.

This scoping review, conducted by a multidisciplinary team of forensic doctors and psychologists with expertise in suicidology, aims to map the state-of-the-art in the detection of suicidal ideation in online posts. Specifically, we seek to: (a) synthesize the main approaches used in the literature, (b) identify key methodological and ethical challenges, and (c) outline directions for future research and intervention design in this emerging field.

## 2. Materials and Methods

This scoping review followed the JBI for Scoping Reviews and it was guided by the following question: “*What is the current state of the art in detecting suicidal ideation in online posts?*”. The question was approached by a multidisciplinary team, composed of forensic doctors and psychologists. No ethical approval was required.

### 2.1. Information Sources and Searching

Following PRISMA-ScR guidelines (Page et al., 2021; Tricco et al., 2018), an initial exploratory search was conducted on PubMed by one researcher (B.T.). The search strategy was then collaboratively refined by two researchers (B.T. and G.B.) and subsequently adopted for use across additional databases. The final search, completed in April 2025, was performed on PubMed, Scopus, and Web of Science using the Boolean string reported in Table 1. Search terms included Medical Subject Headings (MeSH) where applicable. Gray literature was not included. The protocol for this scoping review was registered on the Open Science Framework (OSF; Trentarossi et al., 2025). Given the specific aim of this ScR, terms related to self-harm were intentionally excluded to maintain conceptual clarity and focus.

### 2.2. Study Selection

Following the search strategy, all identified references were imported into Rayyan (Ouzzani et al., 2016), where duplicate records were automatically removed. Two researchers (B.T. and M.E.R.) screened titles and abstracts based on predefined inclusion and exclusion criteria (Table 2), and selected articles for full-text assessment. In cases of uncertainty, a third researcher (G.B.) was consulted to reach a consensus on study inclusion. The selection process, including the number of studies included and excluded at each stage, is reported in accordance with the PRISMA-ScR extension guidelines (Page et al., 2021; Tricco et al., 2018).

### 2.3. Data Extraction and Analysis

One researcher (B.T.) completed the data extraction table for all articles meeting the inclusion criteria. The extracted information included: author, year of publication, journal, purpose of the analysis, major macro-area, type of analysis, platform analyzed, sample characteristics, and main findings. An overview of the key features of the included studies is presented in Appendix A. In order to better understand, a full reading of the texts of the included articles allowed, through an inductive method, to categorize the texts into two major macro-areas, kept throughout the paper and discussed in order to provide an overall view of the phenomenon. More precisely, interpretative studies tried to extract content from the texts in order to understand better what the author was trying to convey, while predictive studies aimed to assess and predict the suicidal risk related to these posts.

## 3. Results

### 3.1. Search Results

**Figure 1:**
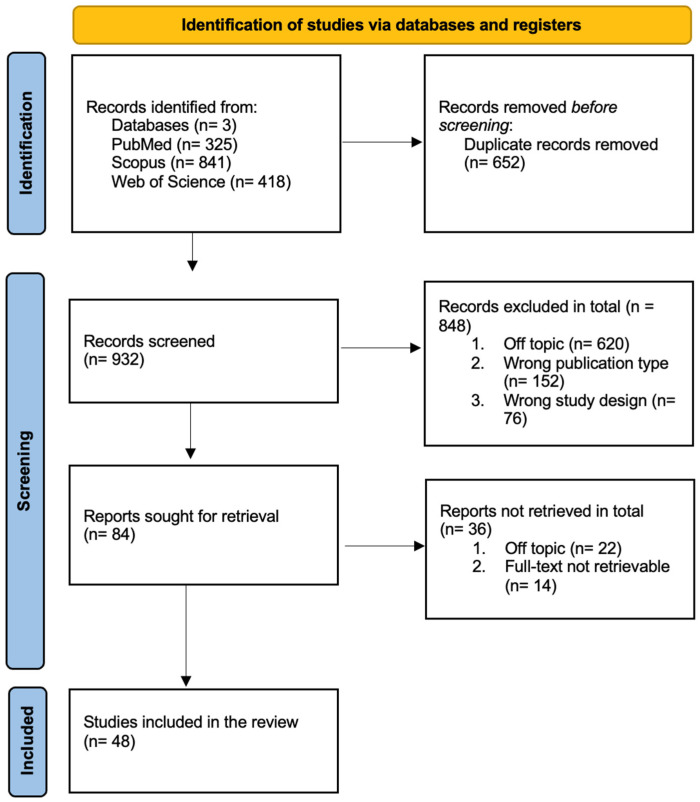
PRISMA flowchart.

A total of 1584 studies were identified through the database search. After the automatic removal of duplicates, 932 unique records remained for title and abstract screening. Based on the predefined inclusion and exclusion criteria, 848 articles were excluded during this stage. Consequently, 84 full-text articles were assessed for eligibility, of which 48 studies met the criteria and were included in the final version of this scoping review. The screening process was conducted independently by two reviewers, with disagreements resolved by discussion or consultation with a third researcher. The complete selection process is illustrated in the PRISMA-ScR flow diagram (Figure 1).

### 3.2. Study Characteristics

The studies were conducted over a period of time from 2009 to 2025, mainly after 2020 (85.42%; n = 41).

The majority of studies were conducted on posts written in English (75%; n = 36) (Cash et al., 2013; Horne & Wiggins, 2009; Mirtaheri et al., 2023; Ali & Gibson, 2019; Carlyle et al., 2018; Beriña & Palaoag, 2024; Lim & Loo, 2023; Lao et al., 2022; Metzler et al., 2022; Ptaszynski et al., 2021; Mobin et al., 2024; Agarwal et al., 2025; Shukla & Singh, 2025; Bouktif et al., 2025; Allam et al., 2025; Abdulsalam et al., 2024; Rezapour, 2023; Grant et al., 2018; Jere & Patil, 2023; Luo et al., 2020; Slemon et al., 2021; Sawhney et al., 2021; Gu et al., 2023; Guidry et al., 2021; Rabani et al., 2023; Anika et al., 2024; Priyamvada et al., 2023; Aldhyani et al., 2022; Chatterjee et al., 2022; Renjith et al., 2022; Saha et al., 2023; Chadha & Kaushik, 2022; Tadesse et al., 2020; Roy et al., 2020; Rabani et al., 2020). One study analyzed posts translated from Portuguese into English (Alghazzawi et al., 2025), four focused on posts in Chinese (J. Liu et al., 2022; X. Liu & Liu, 2021; Meng et al., 2025; Wang et al., 2018), two on posts in Spanish (García-Martínez et al., 2022; Ramírez-Cifuentes et al., 2020) and Thai (Benjachairat et al., 2024; Boonyarat et al., 2024), and the remaining studies involved posts in Arabic (Abdulsalam et al., 2024), Russian (Narynov et al., 2020), and Dutch (Desmet & Hoste, 2018).

Posts were pulled from online platforms, the most common ones were Reddit (Beriña & Palaoag, 2024; Lao et al., 2022; Ptaszynski et al., 2021; Mobin et al., 2024; Bouktif et al., 2025; Alghazzawi et al., 2025; Rabani et al., 2023; Rezapour, 2023; Grant et al., 2018; Jere & Patil, 2023; Slemon et al., 2021; Sawhney et al., 2021; Aldhyani et al., 2022; Renjith et al., 2022; Chadha & Kaushik, 2022; Tadesse et al., 2020) and Twitter (Lim & Loo, 2023; García-Martínez et al., 2022; Metzler et al., 2022; Agarwal et al., 2025; Benjachairat et al., 2024; Allam et al., 2025; Abdulsalam et al., 2024; Boonyarat et al., 2024; Luo et al., 2020; Sarsam et al., 2021; Priyamvada et al., 2023; Saha et al., 2023; Roy et al., 2020; Rabani et al., 2020) followed by Sina Weibo (Wang et al., 2018; X. Liu & Liu, 2021; Meng et al., 2025; Gu et al., 2023; J. Liu et al., 2022) Tumblr (Ali & Gibson, 2019), MySpace (Cash et al., 2013), Instagram (Carlyle et al., 2018), Vkontakte (Narynov et al., 2020) and Pinterest (Guidry et al., 2021). In some cases, posts were taken from multiple platforms (Anika et al., 2024; Desmet & Hoste, 2018; Horne & Wiggins, 2009; Mirtaheri et al., 2023; Rabani et al., 2023; Shukla & Singh, 2025; Ramírez-Cifuentes et al., 2020; Chatterjee et al., 2022).

It was not possible to define the characteristics of the population examined as sex and average age, because in almost all articles, they were not specified.

### 3.3. Purpose of the Analysis

The studies included in this review were classified into two main categories based on their primary aim: interpretative (n = 12) and predictive (n = 36). Although some studies contained elements of both approaches, each was categorized according to its predominant focus. Interpretative studies primarily aimed to explore how suicidal ideation is framed and expressed in online environments. These analyses were interested in the language of users posting suicidal ideation, analyzing rhetorical strategies, jargon, and linguistic cues for authenticity and emotional states. They also investigated the characteristics and attributes of vulnerable users, including posting behaviors and behavioral patterns, to understand the underlying causes of suicidal behavior. In addition, these studies witnessed how online communities react to posts perceived to be indicative of suicide risk, for instance, the encouragement or responses provided by other members (Cash et al., 2013; Horne & Wiggins, 2009; Ali & Gibson, 2019; Wang et al., 2018; Carlyle et al., 2018; Lim & Loo, 2023; Ptaszynski et al., 2021; X. Liu & Liu, 2021; Grant et al., 2018; Guidry et al., 2021; Luo et al., 2020; Slemon et al., 2021).

On the other hand, the primary goal of the predictive studies was to identify posts that may indicate suicidal ideation, distinguish them from neutral posts, and, in some cases, classify them into different levels of suicide risk. These studies are expertise in developing and improving techniques by analyzing linguistic, emotional, and behavioral patterns in user-generated content on social media sites like Twitter, Weibo, Reddit, and others, to detect suicidal ideation early and with accuracy (Mirtaheri et al., 2023; Beriña & Palaoag, 2024; García-Martínez et al., 2022; Lao et al., 2022; Metzler et al., 2022; Mobin et al., 2024; Agarwal et al., 2025; Alghazzawi et al., 2025; Benjachairat et al., 2024; Shukla & Singh, 2025; Bouktif et al., 2025; Allam et al., 2025; Meng et al., 2025; Abdulsalam et al., 2024; Ahadi et al., 2024; Boonyarat et al., 2024; Narynov et al., 2020; Rezapour, 2023; Desmet & Hoste, 2018; Jere & Patil, 2023; Sarsam et al., 2021; Sawhney et al., 2021; Gu et al., 2023; Rabani et al., 2023; Anika et al., 2024; Priyamvada et al., 2023; Aldhyani et al., 2022; Chatterjee et al., 2022; Renjith et al., 2022; Saha et al., 2023; Chadha & Kaushik, 2022; J. Liu et al., 2022; Ramírez-Cifuentes et al., 2020; Tadesse et al., 2020; Roy et al., 2020; Rabani et al., 2020). This category also includes studies aimed at developing artificial intelligence systems for the automated detection of suicide-risk posts.

### 3.4. Type of Analysis

Regarding the methods used to analyze post with suicidal ideation, two main approaches emerged: traditional analysis and artificial intelligence-based analysis. Several studies adopted a combination of both. In these cases, content analysis was first conducted using traditional qualitative or quantitative methods, which was then followed by the development of automated techniques for detecting suicide risk through artificial intelligence.

### 3.5. Traditional Methods

Regarding the analysis methods classified as traditional (n = 8), content analysis was predominantly conducted using qualitative approaches. In particular, three studies applied thematic analysis following the framework developed by Braun and Clarke (Ali & Gibson, 2019; Slemon et al., 2021; Wang et al., 2018). Other qualitative methods included discursive analysis (Horne & Wiggins, 2009) and Rhetorical Structure Theory (RST) (X. Liu & Liu, 2021). One study (Cash et al., 2013) conducted a qualitative analysis but did not specify the method used.

In addition, two studies employed quantitative content analysis (Carlyle et al., 2018; Guidry et al., 2021), focusing on measurable linguistic or thematic features. One study (Ptaszynski et al., 2021) utilized the LIWC (Linguistic Inquiry and Word Count) approach, which, although fully quantitative in its structure, was designed to address a qualitatively driven research question. As such, it can be considered a mixed-methods approach that bridges statistical analysis and interpretive insight.

### 3.6. AI-Based Methods

In studies that applied artificial intelligence to the analysis of post with suicidal ideation, the diversity of methodologies makes it difficult to describe each approach in detail. However, a common thread across these studies is the use of AI techniques—particularly machine learning and deep learning—to train models capable of identifying signs of suicidal ideation in online content. For clarity and consistency, a glossary summarizing the main terminologies and methodological terms used across these studies is provided in Appendix B.

A central component of these approaches is Natural Language Processing (NLP), a subfield of AI focused on enabling computers and algorithms to understand, interpret, generate, and respond to human language. Within this domain, NLP is applied at various stages of the analytical process. The most common applications identified in our review include the following:

Preprocessing. The initial phase—common across all studies—involves preprocessing the text to ensure clarity and consistency for machine interpretation. This step typically includes removing punctuation and standardizing the text through procedures such as lowercasing, tokenization (mainly using Python’s Natural Language Toolkit, NLTK), stopword removal, stemming, lemmatization, and part-of-speech (POS) tagging. These processes aim to reduce textual noise and prepare the data for analysis (Mirtaheri et al., 2023; Beriña & Palaoag, 2024; Lim & Loo, 2023; Lao et al., 2022; Metzler et al., 2022; Mobin et al., 2024; Agarwal et al., 2025; Benjachairat et al., 2024; Bouktif et al., 2025; Allam et al., 2025; Meng et al., 2025; Abdulsalam et al., 2024; Ahadi et al., 2024; Boonyarat et al., 2024; Narynov et al., 2020; Rezapour, 2023; Desmet & Hoste, 2018; Grant et al., 2018; Luo et al., 2020; Sarsam et al., 2021; Sawhney et al., 2021; Gu et al., 2023; Rabani et al., 2023; Aldhyani et al., 2022; Chatterjee et al., 2022; Renjith et al., 2022; Saha et al., 2023; Chadha & Kaushik, 2022; J. Liu et al., 2022; Ramírez-Cifuentes et al., 2020; Tadesse et al., 2020; Roy et al., 2020; Rabani et al., 2020).

Word Embedding. Word embedding techniques represent words as real-valued vectors that capture their contextual, semantic, and syntactic properties. These vectors reflect the degree of similarity between words, allowing models to recognize related meanings based on proximity in vector space (Mirtaheri et al., 2023). Among the most frequently used techniques in the reviewed studies were Word2Vec (Mirtaheri et al., 2023; Narynov et al., 2020; Grant et al., 2018; Anika et al., 2024; Priyamvada et al., 2023; Aldhyani et al., 2022; Renjith et al., 2022; Saha et al., 2023; J. Liu et al., 2022) and GloVe (Anika et al., 2024). An alternative approach found in several studies is TF-IDF (Term Frequency–Inverse Document Frequency), a statistical method that converts text into numerical vectors by emphasizing the relevance of certain words within documents. TF-IDF was used in place of word embeddings in a number of studies (Lim & Loo, 2023; Metzler et al., 2022; Agarwal et al., 2025; Mobin et al., 2024; Bouktif et al., 2025; Ahadi et al., 2024; Rezapour, 2023; Rabani et al., 2023; Aldhyani et al., 2022; Chatterjee et al., 2022; Tadesse et al., 2020; Rabani et al., 2020).

Sentiment Analysis. Sentiment analysis encompasses a range of techniques used to assess the emotional tone of a text and categorize it accordingly. The studies included in this review applied both lexicon-based and machine learning-based approaches. Lexicon-based methods included LIWC (García-Martínez et al., 2022; Gu et al., 2023; Lao et al., 2022; J. Liu et al., 2022), VADER (Lim & Loo, 2023), and SentiWordNet (Jere & Patil, 2023). Machine learning methods frequently involved classifiers such as Support Vector Machines (SVM; Lao et al., 2022; Agarwal et al., 2025; Metzler et al., 2022; Alghazzawi et al., 2025; Benjachairat et al., 2024; Rabani et al., 2023; Chatterjee et al., 2022; Saha et al., 2023; Ramírez-Cifuentes et al., 2020), logistic regression (Alghazzawi et al., 2025; Chatterjee et al., 2022; Rabani et al., 2020; Saha et al., 2023), and random forest models (Lao et al., 2022; Agarwal et al., 2025; Alghazzawi et al., 2025; Allam et al., 2025; Rezapour, 2023; Rabani et al., 2020, 2023; Saha et al., 2023; Chadha & Kaushik, 2022; Ramírez-Cifuentes et al., 2020). Deep learning techniques were also widely adopted, including Long Short-Term Memory networks (LSTM; Agarwal et al., 2025; Benjachairat et al., 2024; García-Martínez et al., 2022; Mirtaheri et al., 2023), bidirectional LSTM (biLSTM; Meng et al., 2025; Sawhney et al., 2021), Convolutional Neural Networks (CNN; Meng et al., 2025), and combined models such as LSTM-CNN (Beriña & Palaoag, 2024; Bouktif et al., 2025; Ahadi et al., 2024; Priyamvada et al., 2023; Aldhyani et al., 2022; Chatterjee et al., 2022; Renjith et al., 2022; Saha et al., 2023; Chadha & Kaushik, 2022; Tadesse et al., 2020). More recent studies also employed BERT (Bidirectional Encoder Representations from Transformers) models (Boonyarat et al., 2024; Bouktif et al., 2025; Jere & Patil, 2023; Metzler et al., 2022; Sawhney et al., 2021; Shukla & Singh, 2025), reflecting a trend toward more advanced, pre-trained language models capable of capturing deeper linguistic context.

### 3.7. Content Analysis Main Results

As reported in several studies (Sarsam et al., 2021; Anika et al., 2024; Priyamvada et al., 2023; Aldhyani et al., 2022; Chatterjee et al., 2022; Renjith et al., 2022; Tadesse et al., 2020; Beriña & Palaoag, 2024), suicidal and non-suicidal posts differ significantly in terms of vocabulary and thematic content. For instance, Beriña, J.M. et al., found that words such as “pain,” “regret,” and “kill” frequently appear in suicidal posts, whereas terms like “happy” and “get better” are more commonly used in non-suicidal content. Similarly, Priyamvada, B. et al., observed that suicidal posts were often characterized by a sense of hopelessness, in contrast to the optimism and positive affect expressed in non-suicidal tweets.

Several studies have also highlighted the frequent use of specific terms such as “kill” and “die” in suicidal content, including Mobin, M.I. et al., Lim, Y.Q. et al., Rabani, S.T. et al., and Abdulsalam, A. et al. In addition, Wang, Z. et al., Lao, C. et al., and Meng, X. et al. noted a marked preference for the use of first-person pronouns in posts reflecting suicidal ideation—an indicator often linked to heightened self-focus and emotional distress.

Temporal patterns in suicidal posting behavior were also explored in several studies. Meng, X. et al. and Chatterjee, M. et al. both found a higher frequency of suicidal posts during nighttime hours. Luo, J. et al. further investigated the timing of posts, revealing that certain themes were more prevalent on weekends, while others were more commonly discussed during the week—suggesting a potential correlation between topic and temporality.

## 4. Discussion

The analysis of online content expressing suicidal ideation has gained significant attention in recent years, largely due to the increasing role of social media in shaping how individuals, especially younger users, communicate emotional distress and seek support. Social platforms like Facebook, Twitter, and Reddit have become spaces where users may articulate suicidal thoughts, sometimes explicitly. This shift toward digital expression has prompted researchers and technology developers to explore ways of monitoring online posts for early signs of suicide risk, with the aim of enabling preventive interventions.

The findings of this scoping review confirm a strong prevalence of predictive approaches within the literature. The primary objective of many studies is to automatically identify posts containing suicidal ideation and to distinguish them from neutral content. In most cases, this is pursued through the development of detection systems based on artificial intelligence, particularly natural language processing (NLP). These systems are often trained on large corpora of social media data and designed to classify content according to degrees of risk. As shown in several studies (Anika et al., 2024; J. Liu et al., 2022; Priyamvada et al., 2023; Tadesse et al., 2020), combining multiple AI techniques—such as machine learning and deep learning—tends to yield better performance than using either traditional text analysis methods or single-model AI approaches. These combinations allow the models to capture more nuanced features in the data, improving sensitivity to linguistic patterns associated with suicidality.

Some studies have employed a mixed-methods design, combining qualitative or interpretive analysis with computational approaches. For example, research by Benjachairat et al., Ramírez-Cifuentes et al., and Rabani et al. used traditional qualitative methods to examine post content and subsequently trained AI models using these findings. This hybrid strategy aims to overcome one of the key limitations of machine learning systems: their difficulty in interpreting sarcasm, metaphor, or implicit emotional cues. As Metzler et al. emphasize, automated tools often struggle to detect empathy, irony, and other forms of complex communication, which are common in posts expressing distress. These limitations underscore the importance of including human judgment in the process, especially when interpreting ambiguous content or culturally specific expressions of suffering. Another challenge is language: most studies in our review examine English-language posts (75%), while a smaller subgroup looks at other languages. This is important since different languages express ideas and nuances differently, and it is worth knowing whether methods based on English posts can practically be applied to other languages. There is a similar problem with platform type since certain platforms limit text length more than others, which could affect the analysis.

Another challenge identified across several studies concerns the uncertain authenticity of the data. While AI tools can be trained to differentiate between suicidal and non-suicidal content (as demonstrated by Beriña et al., Sarsam et al., Anika et al., Priyamvada et al., Aldhyani et al., Renjith et al., Saha et al., Tadesse et al.), the validity of these classifications remains questionable due to the inability to confirm whether the posts truly reflect suicidal intent. Privacy constraints and ethical considerations make it difficult to link the online content to individuals’ real-life trajectories or mental health outcomes, thus complicating the establishment of reliable ground truth for training algorithms.

Moreover, the heterogeneity of platforms, analytical methods, and evaluation criteria across studies makes it difficult to compare results and define best practices. There is currently no consensus on what constitutes acceptable levels of precision, recall, or interpretability in suicide risk detection models. This lack of standardization hinders the development of robust, generalizable tools. As shown in our review, some models rely on lexical features such as keywords or frequency of first-person pronouns (e.g., Chatterjee et al., 2022; Meng et al., 2025), while others focus on sentiment polarity (e.g., Beriña & Palaoag, 2024; Lim & Loo, 2023; Mobin et al., 2024; Rabani et al., 2023). In some cases, researchers have also examined temporal patterns—such as increased posting at night or on weekends (Lao et al., 2022; Luo et al., 2020; Meng et al., 2025)—to enrich the predictive model. However, integrating these diverse indicators into a unified framework remains a substantial methodological challenge.

In recent developments, researchers have begun to explore multimodal approaches that combine textual, visual, and behavioral data to improve detection performance and interpretability. For example, Badian et al. (2023) demonstrated how social media images, when analyzed through large language–vision models, can significantly contribute to predicting suicide risk. Their study highlights the potential of integrating visual cues—such as facial expressions, image content, or symbolic imagery—with linguistic data to form a more holistic understanding of online suicidality. This emerging direction not only enhances model accuracy but also raises important questions about interpretability and ethical boundaries, particularly when personal visual data is involved.

In addition to technical concerns, the deployment of these systems raises significant ethical questions. False positives can result in unnecessary distress or breach of privacy for users flagged as “at risk,” while false negatives may leave genuinely vulnerable individuals without support. Furthermore, without adequate transparency, AI systems risk perpetuating algorithmic bias, especially when trained on datasets that lack demographic diversity or contextual sensitivity. Issues related to user consent, data anonymization, and the right to opt out of monitoring are not consistently addressed across studies, pointing to a gap between technical innovation and ethical governance.

Finally, it is essential to consider the broader implications of detecting suicidal ideation online. If implemented responsibly, such tools could play a valuable role in early intervention and suicide prevention. However, they should not be seen as standalone solutions. As the literature suggests, AI-powered detection systems must be integrated into wider care networks that include clinicians, psychologists, crisis responders, and peer support communities. Their use should be guided by evidence-based protocols and subjected to ongoing evaluation in real-world contexts.

In summary, the potential of digital technologies to identify signs of suicidality in real time represents an important step forward in the field of prevention. Yet, to fully realize this potential, future research must move toward more ethically grounded, interdisciplinary, and culturally responsive approaches. Advances in artificial intelligence must be matched by equal progress in our understanding of human communication, vulnerability, and care (Loch & Kotov, 2025).

This scoping review highlights how the detection of suicidal ideation in online posts is becoming an increasingly relevant area of research at the crossroads of technology, psychology, and public health. The growing body of literature demonstrates the potential of digital traces as early signals of emotional distress, offering the possibility of timely intervention—particularly through the use of artificial intelligence and natural language processing tools. These technologies have shown encouraging results in identifying at-risk content and distinguishing it from neutral or less severe expressions of discomfort.

Despite this progress, the field remains fragmented and marked by significant challenges. Among the most urgent are the lack of standardized and ethically validated datasets, limited cross-cultural applicability of the models, and concerns regarding consent, privacy, and algorithmic bias. Furthermore, the interpretative limitations of current AI systems, such as their difficulty in understanding metaphors, irony, or context-specific meanings, underline the need for hybrid models that integrate machine learning with human judgment.

Moving forward, it is essential to foster stronger interdisciplinary collaboration across fields such as computational science, clinical psychology, linguistics, and forensic medicine. The development of ethically sourced and high-quality training datasets—including, when appropriate, real suicide notes—may help improve the precision and sensitivity of detection systems. However, technological effectiveness alone is not enough. Detection tools must be designed and implemented in ways that are ethically grounded, clinically meaningful, and respectful of the complexity of human suffering.

Despite its strengths, this study also presents a few limitations. For example, only English studies were included, which may have excluded relevant research conducted in other languages. Moreover, the review focused specifically on suicidal ideation, intentionally excluding terms related to self-harm or suicide attempts to maintain conceptual clarity, which may have narrowed the scope of evidence. Future research should aim to overcome these limitations by broadening linguistic and cultural coverage, incorporating non-English studies, and including material from the grey literature.

The digital environment offers a valuable opportunity to reach individuals at risk, often before a crisis fully emerges. But this potential can only be realized through responsible innovation that places care, dignity, and human oversight at the center of technological development. In this sense, detection systems should not only serve as instruments of classification but as tools that contribute to a broader culture of prevention and compassionate engagement.

## Figures and Tables

**Table 1:** Search strategy.

Concept	Keywords/Search Terms
1. Suicidal Ideation	“suicid* ideation” OR “suicide intention” OR “suicid* thoughts”
2. Digital Context	“social media” OR “online post*” OR “internet forum*” OR “digital platform*”
3. Research Activity	“analysis” OR “study” OR “evaluation” OR “assessment” OR “investigation” OR “examination”
4. Final Boolean Search	(“suicid* ideation” OR “suicide intention” OR “suicid* thoughts”) AND (“social media” OR “online post*” OR “internet forum*” OR “digital platform*”) AND (“analysis” OR “study” OR “evaluation” OR “assessment” OR “investigation” OR “examination”)

**Table 2:** Inclusion and Exclusion Criteria.

**Inclusion Criteria**
1. Language: publications in English
2. Studies concerning the analysis of suicidal ideation online
**Exclusion Criteria**
1. Studies not related to online post
2. Studies related only to specific categories
3. Not journal articles
4. Literature reviews, letters or opinion papers

## Data Availability

No new data were created or analyzed in this study. Data sharing is not applicable to this article.

## Appendix A. Author, Year of Publication, Journal, Purpose of the Analysis, Type of Analysis, Sample Characteristics and Main Findings

**First Author, Year, Title**	**Journal**	**Aim**	**Interpretative/** **Predictive**	**Type of Analysis,** **Method**	**Platform,** **N° Post,** **Language**	**Main Results**
Scottye J Cash et al. Adolescent Suicide Statements on MySpace (Cash et al., 2013)	Cyberpsychol Behav Soc Netw	To investigate how teenagers remark on their suicide ideas and plans on MySpace.	Interpretative	Traditional Content analysis mixed method	MySpace 64 English	Content analysis identified key themes in comments related to potential suicidality. More than half of the comments expressed merely suicide ideation and lacked particular context. Breakups and other relationship problems were the second most prevalent theme. Substance abuse and mental health issues were less common but nonetheless important. There were no gender or age-group differences that were statistically significant.
Judith Horne et al. Doing being ‘on the edge’: managing the dilemma of being authentically suicidal in an online forum (Horne & Wiggins, 2009)	Sociol Health Illn	This study looks at how members of an online forum for “suicidal thoughts” develop their authenticity in their introductory postings and how other forum members react to them.	Interpretative	Traditional Discursive analysis	Two forums 329 English	Four practices—narrative framing, getting “beyond” despair, exhibiting logic, and not overtly seeking assistance—are used by participants to establish their authenticity, according to the analysis. Additionally, identities were constructed as being psychologically “on the edge” of life and death in both the original and later entries.
Mirtaheri, S.L. et al. A Self-Attention TCN-Based Model for Suicidal Ideation Detection from Social Media Posts (Mirtaheri et al., 2023)	Expert Systems with Applications	In this research, we offer a novel machine-learning approach that uses deep learning and natural language processing to identify suicidality risk in social media posts.	Predictive	AI-based methods	Reddit and Twitter 278,698 English	The examination identifies that the suicidal social media updates tend to have despair messages, i.e., “want to die,” “feel like,” “need help,” and “don’t know.” The semantic examination suggests that the updates are predominantly associated with sadness, followed by feelings such as joy and anger, pointing towards an affluent emotional atmosphere. Curiously, in spite of having apparently positive wording, the majority of posts with suicidal ideation have been labeled as positive, though in general, their context is negative. In addition, polarity and subjectivity scores show heavy dominance of negative sentiment. These findings can facilitate suicidal ideation detection in early stages using automatic language processing.
Ali A et al. Young People’s Reasons for Feeling Suicidal. (Ali & Gibson, 2019)	Crisis	The purpose of this study was to determine the causes of suicidal thoughts among young people in posts published	Interpretative	Traditional Braun and Clarke’s thematic analysis	Tumblr 210 English	The study identifies the loneliness and social isolation as the risk factors among young people, with a desire for a sense of belonging. Stigma, especially on gender, sexual orientation, and social norms, also contributes to higher suicide rates among marginalized groups. Additionally, self-blame in conjunction with social pressure has a tendency to create feelings of inadequacy and failure. Feelings of helplessness are a shared experience among the majority of young people, without the ability to alter their situation. Though mental illness was recorded less often, a few assigned suicidality to some diagnoses, sometimes as a justification for emotional pain.
Wang Z et al. A Study of Users with Suicidal Ideation on Sina Weibo (Wang et al., 2018)	Telemed J E Health	In order to classify the themes of negative postings published by USI and identify the traits of people who might be at risk of suicide, this study examined whether and how suicidal ideation is discussed on Weibo. It also looked into the possibility of using social media to identify and establish an early warning system for USI.	Interpretative	Mixed Method Braun and Clarke’s thematic analysis and Machine Learning	Sina Weibo 10,537 + 1 million Chinese	The three personal pronouns that were most frequently used were “I,” “you,” and “myself.” The majority of negative posts revealed the causes of their unpleasant emotions, which included fatigue, misunderstanding, and irritation. The second most frequent theme was just expressing sadness. The frequency of the other three motifs was significantly lower. Of the postings, about 2.5% mentioned having a suicidal propensity. Just 1.1% of the posts explained why they were considering suicide.
Carlyle, K. E. et al. Suicide conversations on InstagramTM: contagion or caring? (Carlyle et al., 2018)	Journal of Communication in Healthcare	Studying suicide-related content on Instagram is essential because of the platform’s extensive use and the paucity of research on its effects, which suggests that its possible role in suicide contagion warrants further investigation.	Interpretative	Traditional Quantitative content analysis	Instagram 500 English	The most prevalent graphics were text-based, and the majority of posts mentioned despair (88.4%) and self-harm (60.8%). Posts that talked about self-harm, loneliness, or seeking assistance got a lot more likes. Rarely were WHO standards on suicide contagion adhered to. Engagement was higher for those that expressed suicidal intent or that received encouraging responses.
Beriña M. J. et al. A Natural Language Processing Model for Early Detection of Suicidal Ideation in Textual Data—A Natural Language Processing Model for Early Detection of Suicidal Ideation in Textual Data (Beriña & Palaoag, 2024)	Journal of Information Systems Engineering and Management	Enhancing the automatic detection and reporting of suicide messages is the aim of this research.	Predictive	AI-based methods	Reddit 5000 + 5000 English	The research established clear language differences between suicidal and non-suicidal messages. Suicidal messages included distress- and negative-related words, while non-suicidal messages were positive and optimistic. The trend supported successful classification by AI, with the CNN-LSTM model performing the best and achieving 94% accuracy.
Lim Y.Q. et al. Characteristics of Multiclass Suicide Risks Tweets through Feature Extraction and Machine Learning Techniques (Lim & Loo, 2023)	JOIV International Journal on Informatics Visualization	The application of a machine learning framework that can analyze multiclass categorization of suicide risks from social media postings with in-depth examination of linguistic features that aid in suicide risk identification is suggested in this study.	Interpretative	AI-based methods	Twitter 690 English	Language analysis showed that individual features like PoS tags had no impact, while TF-IDF and sentiment scores were best at differentiating levels of risk. Medium-risk tweets used ideation-related terms, while high-risk tweets used more extreme terms like “kill.” No single feature was sufficient in isolation, but all features combined improved model performance.
García-Martínez C Exploring the Risk of Suicide in Real Time on Spanish Twitter: Observational Study. (García-Martínez et al., 2022)	JMIR Public Health Surveill	The goal was to investigate the emotional content of Spanish-language Twitter posts and how it relates to the degree of suicide risk at the time the tweet was written.	Predictive	Mixed Method	Twitter 2509 Spanish	Out of 2509 tweets, 8.61% were identified as exhibiting suicidality by experts. Suicide risk was highly correlated with emotions like sadness, decreased happiness, overall risk, and intensity of suicidal ideation. Risk intensity was higher in tweets with defeat, rejection, helplessness, desire for escape, lack of support systems, and frequent suicidal ideation. Multivariate analysis yielded intensity of suicidal ideation as a robust predictor of risk severity and emotional components including fear and valence. The model accounted for 75% of the variance.
Lao C et al. Analyzing Suicide Risk From Linguistic Features in Social Media: Evaluation Study. (Lao et al., 2022)	JMIR Form Res.	This project intends to investigate whether computerized language analysis could be used to evaluate and better comprehend an individual’s risk of suicide on social media.	Predictive	AI-based methods	Reddit 866 English	According to the study, at-risk users (low, moderate, and severe) exhibited less clout and more denial, first-person singular pronoun usage, and authenticity than non-risk users. Within at-risk groups, the Kruskal–Wallis test revealed no discernible differences. Strong model performance was demonstrated by the Random Forest model, which excelled at identifying users who were at risk, with AUCs ranging from 0.67 to 0.68. For the model to be accurate, characteristics like negativity and genuineness were crucial. But the SVM model did not perform well, especially for the group that was not at risk.
Metzler H. et al. Detecting Potentially Harmful and Protective Suicide-Related Content on Twitter: Machine Learning Approach. (Metzler et al., 2022)	J Med Internet Res.	This work uses machine learning and natural language processing techniques to categorize vast amounts of social media data based on traits found to be either helpful or detrimental in media effects research on suicide prevention.	Predictive	AI-based methods	Twitter 3202 English	Deep learning models pretrained over large-scale datasets, i.e., BERT and XLNet, outperformed traditional methods in the classification of suicide-related tweets with high F1-scores (up to 0.83 for binary classification). They worked well for suicidal ideation, coping, prevention, and actual suicide cases but could not deal with subjective language like sarcasm and metaphor. Surprisingly, approximately 75% of suicide-related tweets were about actual suicides.
Ptaszynski M. Looking for Razors and Needles in a Haystack: Multifaceted Analysis of Suicidal Declarations on Social Media-A Pragmalinguistic Approach. (Ptaszynski et al., 2021)	Int J Environ Res Public Health.	We examine the terminology used by suicidal users on the Reddit social media network.	Interpretative	Traditional LIWC	Reddit ND English	Its potential applicability was then further analyzed for its limitations, where we found that although LIWC is a useful and easy-to-administer tool, the lack of any kind of contextual processing makes it useless as a tool in psychological and linguistic research. We thus conclude that the shortcomings of LIWC can be easily addressed by creating a number of high-performance AI classifiers trained on annotation of similar LIWC categories, which we plan to investigate in future work.
M. I. Mobin et al. Social Media as a Mirror: Reflecting Mental Health Through Computational Linguistics (Mobin et al., 2024)	IEEE Access	This study uses artificial intelligence and machine learning techniques to examine people’s social media posts in order to assess their mental health, with a focus on indicators that could point to a suicide risk.	Predictive	AI-based methods	Reddit 2016 English	The study shows that suicidal users write longer posts with words like “kill” or “die” and depressed users write short, slang-filled comments. Topics like pessimism and self-harm were detected by models like LDA, Word2Vec, and TF-IDF as major topics, with suicide posts with greater behavioral intensity. Transfer learning showed a high overlap between suicidal ideation and depression, with the need for early detection and intervention.
Agarwal, D. et al. Dwarf Updated Pelican Optimization Algorithm for Depression and Suicide Detection from Social Media. (Agarwal et al., 2025)	Psychiatric Quarterly	By tackling the issues of variability and model generalization, this study offers a unique method for detecting depression and suicide using social media (SADDSM).	Predictive	AI-based methods	Twitter ND English	Using deep learning approaches (DBN, RNN, enhanced LSTM) tuned with the DU-POA algorithm, the SADDSM model obtained great precision (~95%). Because of TF-IDF, improved Word2Vec, and style-based features, it performed better than previous models. The study identified difficulties such as privacy concerns, post-misinterpretation, and high computational needs despite encouraging results. In order to facilitate medical intervention, future study will try to incorporate text, audio, and video data and use models like Random Forest and SVM for multiclass and real-time detection.
Liu X et al. Online Suicide Identification in the Framework of Rhetorical Structure Theory (RST). (X. Liu & Liu, 2021)	Healthcare	The goal was to investigate the rhetorical distinctions between Chinese social media users who did not have suicidal thoughts and those who did.	Interpretative	Traditional Rhetorical Structure Theory (RST)	Online Chinese platform 10,111 + 3740 Chinese	The study contrasted microblog posts from suicide and non-suicide groups (both with 4 men and 11 women). The suicide group contained significantly more posts and rhetorical relations than the non-suicide group. While “Joint” was utilized as the most common rhetorical relation in both groups, variations came in others like “Antithesis,” “Background,” and “Purpose.” When word count was controlled, the differences were only marginally significant.
Alghazzawi D. et al. Explainable AI-based suicidal and non-suicidal ideations detection from social media text with enhanced ensemble technique (Alghazzawi et al., 2025)	Sci Rep.	The work’s goal is to develop a binary-label text classification system that can distinguish between thoughts of suicide and those that are not.	Predictive	AI-based methods	Reddit 12,638 Portuguese translated in English	The study recognized that explicit and personal terminology reflects genuine suicidal ideation and vague or hyperbolic terminology suggests false intent. High precision and good AUC-ROC, especially for false ideation detection, was achieved by an ensemble model boosted with Explainable AI (XAI). The use of multiple classifiers ensured generalizability, while cross-validation and data balancing ensured robustness. The study emphasizes the need to personalize explanations to users and retrain models to comply with changing language trends.
Benjachairat, P. et al. Classification of suicidal ideation severity from Twitter messages using machine learning (Benjachairat et al., 2024)	International Journal of Information Management Data Insights	Text categorization models for predicting the intensity of suicidal thoughts are presented in this study.	Predictive	Mixed Method	Twitter 20,138 Thai	The study employs Twitter data to train a machine learning model that recognizes suicidal ideation levels, to assist the CHUOSE app, using CBT principles to process user messages. While not a replacement for clinical therapy, the app may help with treatment and be guided by clinicians as a therapeutic tool. Context understanding and coverage of CBT should be enhanced.
Shukla, S.S.P. et al. Enhancing suicidal ideation detection through advanced feature selection and stacked deep learning models (Shukla & Singh, 2025)	Applied Intelligence	Since social media and other communication platforms are often used for emotional expression and might reveal notable behavioral changes, it is essential for suicide prevention to identify suicidal ideation on these platforms.	Predictive	AI-based methods	Twitter and Reddit 30,816 Reddit + 9119 Twitter English	The hybrid approach proposed is 97% accurate on Twitter data, outperforming previous models. PBFS feature selection in conjunction with BERT, LLM, and MUSE embeddings for enhanced semantic understanding is employed. Stacked architecture from BiGRU-Attention, CNN, and XGBoost boosted the classification performance. The model generalized well but has the drawback of being text-based only, and multimodal work needs to be explored in the future.
Bouktif S. et al. Explainable Predictive Model for Suicidal Ideation During COVID-19: Social Media Discourse Study (Bouktif et al., 2025)	J Med Internet Res.	Using machine and deep learning algorithms on social media data, this study sought to find trends and indications linked to suicide thoughts.	Predictive	AI-based methods	Reddit 1338 + 1816 English	The study examined 3154 Reddit posts, of which 42.4% were tagged suicidal. A BERT + CNN + LSTM model reported 94% precision, 95% recall, and 93.65% accuracy with robust performance validated through 10-fold cross-validation. Analysis showed significant shifts in suicidal language across COVID-19, with XAI tools (LIME and SHAP) identifying salient terms such as “COVID,” “life,” and “die” as robust predictors of suicidal ideation.
Allam H. et al. AI-Driven Mental Health Surveillance: Identifying Suicidal Ideation Through Machine Learning Techniques (Allam et al., 2025)	Big Data and Cognitive Computing	The article’s goal is to use internet language as a monitoring and early intervention tool while utilizing AI and machine learning to enhance suicide prevention.	Predictive	AI-based methods	Twitter 20,000 English	The model achieved 85% accuracy, 88% precision, and 83% recall in identifying suicidal posts. It picked up notable emotional markers like “hurt,” “lost,” and “better without.” The balanced performance was 0.85 for F1-score and 0.93 for PR-AUC, but higher recall could capture more severe cases.
Meng, X. et al. Mining Suicidal Ideation in Chinese Social Media: A Dual-Channel Deep Learning Model with Information Gain Optimization (Meng et al., 2025)	Entropy	Using a fine-grained text enhancement technique, our model is made to process Chinese data and grasp the subtleties of text locale, context, and logical structure.	Predictive	AI-based methods	Sina Weibo ND Chinese	The study found that BERT embedding deep learning models outperform traditional models in suicidal ideation identification on Chinese social media. Combining information gain and attention mechanisms promoted focus on significant indicators, whereas small convolution kernels enhanced identification and reduced complexity. Linguistic analysis proved that suicidal users utilize more verbs and first-person pronouns, which aligns with the success of the model.
Abdulsalam, A. et al. Detecting Suicidality in Arabic Tweets Using Machine Learning and Deep Learning Techniques (Abdulsalam et al., 2024)	Arab J Sci Eng	Use machine learning and deep learning techniques to automatically categorize tweets in Arabic that contain suicide thoughts (as opposed to non-suicidal ones).	Predictive	AI-based methods	Twitter 5719 Arabic	According to the study, several terms associated with death and self-harm were frequently used in suicidal tweets, including “I want to die” and “I will kill myself.” Usually, these tweets were between two and twenty words long. The language employed was characterized by emotional turmoil and a clear intention to damage oneself. Furthermore, the number of suicidal tweets decreased around 5 PM after peaking at around 10 PM. These tweets’ timing and content provide important information for enhancing suicide ideation detection systems.
Ahadi S.A. et al. Detecting suicidality from Reddit Posts Using a Hybrid CNN-LSTM Model (Ahadi et al., 2024)	JUCS—Journal of Universal Computer Science	A multi-layered classification model that combines a convolutional neural network (CNN) and a long short-term memory (LSTM) network is used in this paper to propose an integrated framework for identifying suicidal ideation on social media.	Predictive	AI-based methods	Reddit 59,996 English	The study designed a hybrid CNN-LSTM classifier to detect suicidal ideation in text on social media. There was enhanced accuracy, robustness, and reduced overfitting with stacked and bagging ensembles. The best performance was obtained using TF-IDF embeddings, RReLU activation, and the Adam optimizer, boosting performance by up to 15%. Further study is needed to tackle data imbalance, model interpretability, and ethical issues.
Boonyarat, P. et al. Leveraging enhanced BERT models for detecting suicidal ideation in Thai social media content amidst COVID-19 (Boonyarat et al., 2024)	Information Processing and Management	The study’s two goals were to identify emotions (ER) and detect suicidal ideation (SID) via binary classification (whether suicidal ideation was present in tweets or not).	Predictive	AI-based methods	Twitter 2400 Thai	The model detected excessive suicidal ideation and negative emotions in Thai Twitter data, indicating long-standing psychological distress after the COVID-19 pandemic. Results show that there are long-term mental health effects from pandemic lockdowns with frustration, hopelessness, and emotional suffering seen among users. Findings are consistent with international trends and emphasize the need for continuous monitoring of mental health. Restrictions include no ground truth and data limited to Twitter with future intent to expand to other platforms.
Narynov, S. et al. Applying natural language processing techniques for suicidal content detection in social media (Narynov et al., 2020)	Journal of Theoretical and Applied Information Technology	The goal of this project is to create and assess computer techniques for identifying and assessing depressive and suicidal content in texts from social networks.	Predictive	AI-based methods	Vkontakte microblogging platform ND Russian	Texts were lemmatized and stop-words eliminated prior to vectorization using TF-IDF and Word2Vec. Various classifiers were trained, the optimum result being produced by the Random Forest classifier using TF-IDF at 96%. ROC curve validation and cross-validation were able to establish its accuracy in labeling suicidal and depressive posts.
Rezapour, M. Contextual evaluation of suicide-related posts. (Rezapour, 2023)	Humanit Soc Sci Commun	The purpose of this study was to find mental stresses associated with suicide and investigate the posts’ contextual characteristics, particularly those that are incorrectly labeled.	Predictive	AI-based methods	Reddit 22,498 English	Our findings suggest that while machine learning algorithms can identify most of the potentially offending posts, they can also introduce bias and some human touch is required to minimize the bias. We feel that there will be some posts that will be very difficult or impossible for algorithms to accurately label on their own, and they do require human empathy and insight.
Desmet, B. et al. Online suicide prevention through optimised text classification (Desmet & Hoste, 2018)	Information Sciences	This study aims to investigate supervised text classification to model and detect suicidality in Dutch-language forum posts in alternative to keyword search methods, typically used online	Predictive	AI-based methods	Dutch-language forum ND Dutch	The study revealed that automatic text classification can efficiently be applied to classify suicide and high-risk content with great accuracy, something that can be applicable for prevention in real-life scenarios. While recall was excellent in detecting relevant content, it was low in severity, especially due to implicit reference to suicide. Optimization using feature selection and genetic algorithms improved model performance. However, the reliance of the system on labeled data as well as its Dutch nature are significant limitations. Future projects involve text normalization and cross-lingual transfer to enhance recall and broaden applicability.
Grant RN et al. Automatic extraction of informal topics from online suicidal ideation. (Grant et al., 2018)	BMC Bioinformatics.	By examining social media posts and identifying at-risk individuals using expert-derived indicators and language traits, the study seeks to understand factors connected to suicide.	Interpretative	AI-based methods	Reddit 131,728 English	The cluster analysis was able to identify key themes of suicidal thoughts, self-injury, and abuse that mapped onto expert-identified risk factors. More specific risks like drug abuse and stressors like school pressure, body image, and medical issues were also revealed, though some, like gun ownership, were less represented.
Jere S. et al. Detection of Suicidal Ideation Based on Relational Graph Attention Network with DNN Classifier (Jere & Patil, 2023)	International Journal of Intelligent Systems and Applications in Engineering	In order to detect suicidal ideation by analyzing Twitter posts, a model based on a Relational Graph Attention Network (RGAT) in conjunction with a DNN classifier will be developed and evaluated. This model will integrate natural language processing techniques to identify at-risk individuals early and outperform current models.	Predictive	AI-based methods	Reddit 233,372 English	The study proved that automatic text classification can accurately pinpoint suicide-related content and can be used for prevention. While high recall was established on relevant posts, it was low on severity due to implicit references. Feature selection and genetic algorithms increased performance. The limitations are based on labeled data and Dutch focus. Future work aims to increase recall via cross-lingual transfer and normalization of the text.
Luo J Exploring temporal suicidal behavior patterns on social media: Insight from Twitter analytics. (Luo et al., 2020)	Health Informatics J.	1. Content analysis was used to identify suicide risk variables in tweets; 2. Suicidality was quantitatively measured to identify temporal patterns of suicidal behavior; and 3. A method for examining temporal patterns of suicidal behavior on Twitter was proposed.	Predictive	AI-based methods	Twitter 716,899 English	The study identified 13 latent suicide-related topics using NMF topic modeling in tweets. Depression, school, low mood, and being lonely were some of the significant risk factors. Weekend suicide-related activity was seen for a few topics and Monday/Tuesday for others by temporal analysis. Every topic showed distinct weekly patterns, substantiating suicidality as a time-varying concept. These results could be applied in formulating targeted suicide prevention intervention based on behavioral patterns.
Slemon A. et al. Reddit Users’ Experiences of Suicidal Thoughts During the COVID-19 Pandemic: A Qualitative Analysis of r/Covid19_support Posts. (Slemon et al., 2021)	Frontiers in Public Health	To investigate the ways in which members of the r/COVID19_support group on Reddit talk about having suicidal thoughts during the COVID-19 pandemic.	Interpretative	Traditional Braun and Clarke’s thematic analysis	Reddit 83 English	The study determined that employment and financial concern, pre-existing mental illness, and isolation—each heightened by the pandemic—amplified suicidal ideation. Despite online relationships, one still felt isolated. The semi-anonymity of Reddit allowed open talk of mental illness, suggesting possible facilitated integration, but conscientious implementation is required.
Sarsam, S. M. et al. A lexicon-based approach to detecting suicide-related messages on Twitter (Sarsam et al., 2021)	Biomedical Signal Processing and Control	This study investigated how emotions in Twitter posts can be used to identify information related to suicide.	Predictive	AI-based methods	Twitter 4987 English	The study identified that suicide tweets were more angry, sad, and negative in sentiment, while non-suicide tweets were more angry and cheerful. The YATSI classifier, using a semi-supervised approach, was better than the existing methods with high accuracy and 94% agreement with expert labels, indicating improved detection of suicide tweets.
Sawhney R et al. Robust suicide risk assessment on social media via deep adversarial learning. (Sawhney et al., 2021)	Journal of the American Medical Informatics Association	To enhance the model’s capacity for generalization, we provide Adversarial Suicide assessment Hierarchical Attention (ASHA), a hierarchical attention model that uses adversarial learning.	Predictive	AI-based methods	Reddit 500 English	The study finds that ASHA outperforms deep and standard models in being able to correctly detect temporal trends and varied risk levels. Adversarial training regularizes and reduces overfitting. All the model components—ordinal loss, sequential modeling, and adversarial learning—are working towards improved performance. ASHA also picks up on subtle suicidal signals such as misspellings and indirect speech and is more human-judgment-like than previous models.
Gu Y et al. Suicide Possibility Scale Detection via Sina Weibo Analytics: Preliminary Results. (Gu et al., 2023)	Int J Environ Res Public Health	In order to forecast four aspects of the Suicide Possibility Scale—hopelessness, suicidal thoughts, poor self-evaluation, and hostility—we developed a suicide risk-prediction model using machine-learning algorithms to extract text patterns from Sina Weibo data.	Predictive	AI-based methods	Sina weibo 37,474 English	The research employed 121 linguistic features to forecast four SPS dimensions, most of which were taken from SCLIWC and the Moral Foundations Dictionary. The model was found to have satisfactory reliability for Hopelessness (0.72) and Hostility (0.81), with moderate predictive validity of approximately 0.35 Pearson correlation. Overall, it proved satisfactory predictive power through linguistic analysis of Weibo posts.
Guidry JPD et al. Pinning Despair and Distress—Suicide-Related Content on Visual Social Media Platform Pinterest. (Guidry et al., 2021)	Crisis.	This study’s goal was to examine Pinterest posts on suicide.	Interpretative	Traditional quantitative content analysis	Pinterest 500 English	Most suicide-related Pinterest posts were from individuals and were more interactive than organizational posts. Expression of identification as a person who has suicidal thoughts was common in comments, with more than half positive but 24.1% negative or bullying. WHO guideline information was more common in images than in captions but generated less interaction. Emotional and informational support were mostly conveyed in images, though only instrumental support, though infrequent, was linked to higher interaction.
Rabani, S.T. et al. Detecting suicidality on social media: Machine learning at rescue (Rabani et al., 2023)	Egyptian Informatics Journal	The aim of this research is to create a multi-class machine learning classifier to recognize varying degrees of suicidal risk in social media messages.	Predictive	AI-based methods	Twitter and Reddit 19,915 English	The study found that the XGB-EFASRI model performed best in identifying suicidal ideation (accuracy of 96.33%) among other machine learning models by capturing both linear and nonlinear patterns of features. Content analysis revealed that high-risk posts contained explicit suicidal intent and extreme despair, emotional distress but without explicit suicidal language in moderate-risk posts, and no-risk posts included general awareness or information. Topic modeling reinforced obvious semantic variations by risk level, with topics such as loneliness, emptiness, and hopelessness being characteristic of high-risk material.
Anika, S et al. Analyzing Multiple Data Sources for Suicide Risk Detection: A Deep Learning Hybrid Approach (Anika et al., 2024)	International Journal of Advanced Computer Science and Applications	This study’s goal is to automatically identify suicide risk in user-generated content from social media sites by utilizing Natural Language Processing (NLP) and Sentiment Analysis techniques, especially through sophisticated deep learning models.	Predictive	AI-based methods	Twitter and Reddit 241,193 English	Content analysis revealed differential linguistic differences in suicidal and non-suicidal posts, depicted as word clouds using common words. Skip-Gram word embeddings captured informative word relationships best. The BiGRU-CNN model performed better than others in suicidality prediction with good accuracy on both the Reddit (93.07%) and Twitter (92.47%) corpora. The model could effectively capture emotional and contextual information in text with good generalizability across platforms. Deep learning algorithms surpassed the traditional machine learning classifiers consistently in handling the nuance of suicidal content’s language.
Priyamvada, B. Stacked CNN—LSTM approach for prediction of suicidal ideation on social media (Priyamvada et al., 2023)	Multimedia Tools and Applications	The goal of the study is to determine which machine learning and deep learning models are more effective at identifying suicidal ideation signals in Twitter messages.	Predictive	AI-based methods	Twitter 5126 + 4833 English	Content analysis revealed clear lexical divergence between suicidal and non-suicidal tweets, where suicidal tweets showed repetitive use of question marks and despair, while non-suicidal tweets showed positivity and joy. The classification phase compared a number of machine learning models, where XGBoost outperformed other traditional methods with ~89% accuracy. Deep learning models using Word2Vec embeddings were also tried, and it was observed that the Stacked CNN—2 Layer LSTM model provided the highest accuracy of 93.92%. The hybrid CNN-LSTM architecture helped mitigate overfitting and improve stability and prediction capability. Overall, the coupling of CNN and LSTM models was observed to be superior to their individual use.
Aldhyani, T.H.H. et al. Detecting and Analyzing Suicidal Ideation on Social Media Using Deep Learning and Machine Learning Models (Aldhyani et al., 2022)	International Journal of Environmental Research and Public Health	The aim of this work is to propose an experimental research-based approach to create a system for detecting suicidal thoughts.	Predictive	AI-based methods	Reddit 232,074 + 116,037 English	The study analyzed Reddit posts to predict suicidal ideation using textual characteristics and psychometric LIWC-22 characteristics. Posts indicating suicidal ideation had higher scores on authenticity, anxiety, depression, negativity, and despondency, whereas non-suicidal posts had higher scores on attention, perception, sociality, and cognitive processes. The CNN–BiLSTM model achieved 95% using textual characteristics, outperforming XGBoost, while XGBoost outperformed CNN–BiLSTM with LIWC characteristics. Statistical testing confirmed that the individuals who are at risk of suicide have clear indications of psychological distress. Word cloud visualization portrayed the most common repeating words expressing suicidal ideation.
Chatterjee, M. et al. Suicide ideation detection from online social media: A multi-modal feature based technique (Chatterjee et al., 2022)	International Journal of Information Management Data Insights	The primary goal of this study is to assess online social media in order to enable early diagnosis of suicidal thinking.	Predictive	AI-based methods	Twitter and Reddit 188,704 English	Emotional, linguistic, and temporal features were identified as important predictors of suicidal ideation on social media by the research. The individuals posting suicidal ideation were frequently sad, anxious, depressed, and used more negative language and emoticons. Temporal analysis revealed that 73% of the suicidal users were active between 6 p.m. and 6 a.m., which in turn suggests that nighttime use was correlated with depressive states of mind. Sentiment, use of emoticons, and personality were systematically mined out of each post to identify implicit emotional signals. Logistic Regression had the maximum accuracy for classification followed by SVM when all the features that were mined were used.
Renjith, S. et al. An ensemble deep learning technique for detecting suicidal ideation from posts in social media platforms (Renjith et al., 2022)	Journal of King Saud University—Computer and Information Sciences	The goal of the project is to develop a mixed deep learning model that will enhance the automatic classification of Reddit texts that contain suicide thoughts. The posts are analyzed using LSTM, CNN, and an attention mechanism (Attention).	Predictive	AI-based methods	Reddit 69,600 English	Severe Risk posts often utilized negative words, self-referential words, and death words, while No Risk posts contained more positive words. Word cloud and n-gram frequency visualized the distinctions between the postings.
Saha, S. et al. An Investigation of Suicidal Ideation from Social Media Using Machine Learning Approach (Saha et al., 2023)	Baghdad Science Journal	This study’s goal is to use machine learning to assess factors of suicidal behavior in people.	Predictive	AI-based methods	Twitter ND English	The study compared different machine learning models to determine suicidal ideation from social media posts. SVM yielded the maximum accuracy (88.6%), and Gaussian Naive Bayes yielded the highest precision (77.3%). Suicidal posts had more despair-related words, self-injury-related words, and hopelessness-related words, and non-suicidal posts had more positive words.
Chadha, A. A Hybrid Deep Learning Model Using Grid Search and Cross-Validation for Effective Classification and Prediction of Suicidal Ideation from Social Network Data (Chadha & Kaushik, 2022)	New Generation Computing	The goal of this study is to create an effective learning model that can recognize people who are displaying suicide thoughts by precisely evaluating social media data.	Predictive	AI-based methods	Reddit 10,000 + 10,000 English	The authors employed 20,000 Reddit posts for suicidal vs. non-suicidal content classification. The authors, after preprocessing with lemmatization and word vectorization, tried CNN, CNN-LSTM, and CNN-LSTM with attention (ACL). The optimal F1 score of 90.82% and precision of 87.36% were achieved using ACL with GloVe embedding and hyperparameter tuning, while the maximum recall of 94.94% was achieved using the random embedding version with the minimum number of missed suicidal posts.
Liu, J. et al. Detecting Suicidal Ideation in Social Media: An Ensemble Method Based on Feature Fusion (J. Liu et al., 2022)	International Journal of Environmental Research and Public Health	The final objective is to use a hybrid technique that integrates linguistic, psychological, and semantic data to develop an efficient and customized model that can correctly identify post-suicidal ideation.	Predictive	AI-based methods	Weibo 2272 + 37,950 Chinese	The experiment showed that the characteristics of suicide risk factors were optimal for predicting suicidal ideation in Weibo posts with an F1-score of 72.77%. Merging different aspects of content—particularly basic statistics, suicide-related words, and word embeddings—also improved performance. The best model using content (BSC + RFS + WEC) had an accuracy of 80.15% and an F1-score of 78.60% after feature selection, which eliminated noise and highlighted key patterns.
Ramírez-Cifuentes, D et al. Detection of suicidal ideation on social media: Multimodal, relational, and behavioral analysis (Ramírez-Cifuentes et al., 2020)	Journal of Medical Internet Research	Suicide risk assessment was the main application of this study, which investigated the identification of mental health issues on social media.	Predictive	Mixed Method	Twitter 98,619 English	The study emphasizes the necessity of text features to measure suicide risk on social media. Through manually constructed suicide-specific vocabulary (SPV), researchers improved model accuracy by eliminating meaningless text. Self-references, posting frequency, and time intervals between posts were strong predictors (*p* < 0.001). Models trained on generic control users performed better than models that used controls with similar suicidal content. Merging text analysis with relational, behavioral, and image data provided more accurate and understandable predictions.
Tadesse, M.M. et al. Detection of suicide ideation in social media forums using deep learningtade (Tadesse et al., 2020)	Algorithms	The project aims to compare a hybrid prediction model based on deep learning (LSTM + CNN) with more conventional machine learning models in order to detect suicidal ideation in Reddit postings.	Predictive	AI-based methods	Reddit 3549 + 3652 English	The study contrasted frequent n-grams of suicide-suggestive and non-suicidal postings through examination of Reddit postings, and the outcome signaled hopelessness, anxiousness, guilt, and self-focus in suicidal postings and positivity and social interaction in non-suicidal postings. Rhetorical questions and death words signaling distress were prevalent in suicidal postings. The classification experiments signaled that deep learning models, specifically an LSTM-CNN hybrid model that made use of word embeddings, did better than standard machine learning baselines. This model achieved maximum accuracy (93.8%) and F1 score (92.8%) in suicidal ideation detection. Tunings of the features and parameters, such as ReLU activation and max-pooling, also assisted the model in its performance.
Roy, A. et al. A machine learning approach predicts future risk to suicidal ideation from social media data (Roy et al., 2020)	npj Digital Medicine	By examining publicly available Twitter data, the aim was to create an algorithm known as the “Suicide Artificial Intelligence Prediction Heuristic (SAIPH)” that can forecast the likelihood of suicidal thoughts in the future.	Predictive	AI-based methods	Twitter 283 + 3,518,494 English	Neural network models correctly predicted various psychological states with over 70% AUC. Using tweets, they detected suicidal ideation (SI) events with high accuracy (up to 0.90 AUC) and were better than sentiment analysis. Models also detected individuals who had a history of suicide attempts or plans and were stable across sex and age groups. Temporal patterns revealed risk scores are highest surrounding SI events, which suggests early warning potential. Regional SI scores were highly correlated with county-level suicide rates, particularly among youths.
Rabani, S.T. et al. Detection of suicidal ideation on Twitter using machine learning & ensemble approaches (Rabani et al., 2020)	Baghdad Science Journal	In order to help with the early detection and prevention of this severe mental health condition, this research suggests a methodology and experimental strategy that makes use of social media to more thoroughly analyze suicidal ideation.	Predictive	AI-based methods	Twitter 18,756 English	Content analysis was guided by mental health professionals who categorized tweets as suicidal or non-suicidal depending on words and context. Tweets with at least 75% inter-annotator agreement were used to ensure annotation quality. Feature extraction used TFIDF, and lesser important features were removed using information gain. The highest-performing model, Random Forest, recorded a 98.5% accuracy rate in detecting suicidal content.

## Appendix B. Glossary for Readers Related to Main Terminology Used in AI Qualitative Analysis

**Preprocessing**	the stage of preparing and cleaning raw data—for example, by removing errors, missing values, or noise—to make it ready for analysis or use in machine learning models.
**Lowercasing**	process of converting all letters in a text to lowercase
**Tokenization**	process used to divide text into smaller units of information, such as words, phrases, or symbols.
**Stopword removal**	process of eliminating common words that provide little meaningful information.
**Stemming**	the reduction of words to their root or base form, usually by removing suffixes and sometimes prefixes.
**Lemmatization**	process applied to words to transform them into a base or dictionary representation known as a lemma, through the application of linguistic rules.
**Part-of-speech**	categorization of words in a text into their grammatical categories, such as nouns, verbs, and adjectives.
**Word embedding**	represent words as real-valued vectors that capture their contextual, semantic, and syntactic properties. These vectors reflect the degree of similarity between words, allowing models to recognize related meanings based on proximity in vector space.
**TF-IDF**	statistical method that converts text into numerical vectors by emphasizing the relevance of certain words within documents
**Sentiment analysis**	process of detecting and categorizing opinions or emotions expressed in text, usually as positive, negative, or neutral.
**LIWC**	tool for text analysis that quantifies the psychological, emotional, and structural elements of language through predefined word categories.
**Vader**	rule-based sentiment analysis tool designed for social media text that combined a lexicon with intensity measures to capture the polarity of sentiment.
**Senti-word-net**	lexical resource in which each WordNet synset gets numerical scores for positive, negative, and neutral sentiments.
**LSTM**	type of recurrent neural network designed to capture long-term dependencies in sequential data
**CNN**	neural network architecture that uses convolutional layers to extract hierarchical features from data
**LSTM-CNN**	hybrid neural network that combines the LSTM layer for capturing sequential dependencies within text with the CNN layers for extracting feature patterns of a local nature.
**BERT**	transformer-based language model that uses both left and right contexts to generate contextualized word embeddings.
